# New discriminant score to predict the fibrotic stage of non-alcoholic steatohepatitis in Japan

**DOI:** 10.1007/s12072-014-9605-x

**Published:** 2015-01-22

**Authors:** Yusuke Kawamura, Kenji Ikeda, Yasuji Arase, Yushi Sorin, Taito Fukushima, Hideo Kunimoto, Tetsuya Hosaka, Masahiro Kobayashi, Satoshi Saitoh, Hitomi Sezaki, Norio Akuta, Fumitaka Suzuki, Yoshiyuki Suzuki, Hiromitsu Kumada

**Affiliations:** 1 Department of Hepatology, Toranomon Hospital, 2-2-2, Toranomon, Minato-ku, Tokyo, 105-8470 Japan; 2 Okinaka Memorial Institute for Medical Research, Toranomon Hospital, Tokyo, Japan

**Keywords:** Non-alcoholic fatty liver disease, Discriminant score, Fibrosis

## Abstract

**Background and aim:**

Currently, non-alcoholic steatohepatitis (NASH) can only be diagnosed histopathologically. Our objective was to establish a new scoring system for the fibrotic stage of NASH.

**Methods:**

We enrolled 139 patients with histologically proven NASH and divided them into two groups to construct (*n* = 90) and validate (*n* = 49) a fibrotic score for NASH (FSN). We used 17 variables and their natural logarithmic transformations in the multivariate analysis. To assess the accuracy of the FSN in determining NASH advanced fibrosis (stages 3–4), we compared various fibrotic scores for NASH.

**Results:**

In the construct group, multivariate regression analysis ultimately obtained the following function: *z* = 1.022 × ln (type IV collagen 7S) (ng/mL) − 0.00680 × (platelet count) (×10^9^/L) + 1.925 × ln (AST) (IU/L) − 1.239 × ln (ALT) (IU/L) + 0.249. Median values of the FSN for stages 1, 2, 3 and 4 were 1.87, 2.14, 3.26 and 3.89, respectively. The multiple regression coefficient and coefficient of determination were 0.70 and 0.46, respectively. In the validation group, the median value was 2.00, 2.83, 3.08 and 4.37 in each stage. With regard to the utility of the FSN for predicting advanced fibrosis of NASH (stage ≥3), the area under the receiver operating characteristic curves (AUROC), 0.909 (95 % CI 0.847–0.970, *p* < 0.001), was higher than that for the other fibrotic scores (APRI, NAFLD fibrosis score, FIB-4 index, BARD score, NIKEI) in the construct group.

**Conclusions:**

This simple scoring system accurately predicts fibrotic stage and discriminates patients with advanced fibrosis of NASH.

## Introduction

Nonalcoholic fatty liver disease (NAFLD) is a common cause of chronic liver disease in Western countries [1–4], and more recently, in many Asian nations [5, 6]. In particular, patients with nonalcoholic steatohepatitis (NASH), a subcategory of NAFLD, are at increased risk for developing hepatocellular carcinoma [7]. Patients with NAFLD and advanced fibrosis have a higher risk of hepatocarcinogenesis, similar to individuals with viral hepatitis [8–10]. Currently, NASH can only be diagnosed by histopathology. Usually, chronic liver disease is definitively diagnosed from histopathological examination of a biopsy specimen.

The number of patients with NAFLD is expected to increase. In actuality, ultrasonography (US) alone is being used to identify many patients with NAFLD to avoid an invasive histological diagnosis. Therefore, because of increased cost, possible risks (risk of bleeding, allergic reaction caused by local anesthetics, advanced age), and health-care resource utilization, invasive liver biopsy is poorly suited as a diagnostic method for such a prevalent condition. Furthermore, the NASH lesions are unevenly distributed throughout the liver parenchyma; therefore, liver biopsy has inherent sampling errors, which can lead to substantial inaccuracies in stratification and staging [11].

Because of the problems with biopsy for evaluating patients with liver disease, noninvasive diagnostic tools that are not based on image analysis and are easy to implement for outpatient medical care have been used. These include the following: the aspartate aminotransferase (AST)-to-platelet ratio index (APRI) [12], the NAFLD fibrosis score [13], the FIB-4 index [14], the BARD score [15], and the non-invasive Koeln-Essen-index (NIKEI) [16]. The APRI was developed for the prediction of significant fibrosis in patients with chronic hepatitis C [12], and its utility for patients with NAFLD has also been reported [17]. The NAFLD fibrosis score was developed for the prediction of significant fibrosis in patients with NAFLD, and this score is obtained through a formula which includes six variables: age, presence of impaired fasting glucose (IFG) or diabetes, BMI, AST/ALT ratio, platelet count, and albumin. The FIB-4 index was developed as a noninvasive panel for staging liver disease in patients with human immunodeficiency virus/hepatitis C virus (HCV) coinfection [14]. It is based on patient age and values for AST, ALT, and platelet count, which are routinely measured and thus available for virtually all patients with liver disease. This index has also been independently validated in subjects with HCV infection alone [18]. It was recently demonstrated that its performance characteristics for the diagnosis of advanced fibrosis in NAFLD are better than those of other similar noninvasive diagnostic panels [19]. The BARD score (which includes the following 3 variables: body mass index [BMI], aspartate aminotransferase [AST]/alanine aminotransferase [ALT] ratio, and diabetes) is a noninvasive system that was developed to predict advanced fibrosis in patients with NALFD [15]. The NIKEI is a noninvasive system that was developed more recently to predict advanced fibrosis in patients with NALFD [16].

These noninvasive scoring systems perform well for predicting the advanced fibrosis of NASH (approximate positive predictive value [PPV], 43–90 %; negative predictive value [NPV], 83–98 %). However, the usefulness of the discriminant functions was less valuable up to the present time for the following reason: These noninvasive scoring systems were made for the purpose of discriminating severe hepatic fibrosis from mild fibrosis, and they are not intended for subdivision of histological classifications (stages 1, 2, 3 and 4).

In this study, we tried to generate a function to estimate the fibrotic stage of NASH that was objectively diagnosed by liver biopsy. The purpose of this study was, therefore, to develop a reliable multiple regression function and to obtain practical coefficients for significant variables.

## Patients and methods

### Study population

From January 1980 to December 2013, 148 patients were diagnosed with NASH based on histopathological evaluation at Toranomon Hospital, Tokyo, Japan; 139 of these patients were enrolled in this retrospective study. Inclusion criteria were the following: (1) daily alcohol intake of <20 g/day; (2) no underlying viral hepatitis, autoimmune hepatitis, drug-induced liver disease, or primary biliary cirrhosis; (3) no underlying systemic autoimmune diseases, such as systemic lupus erythematosus and rheumatoid arthritis; (4) no underlying metabolic diseases, such as hemochromatosis, alpha-1-antitrypsin deficiency, and Wilson disease; and (5) NAFLD activity score ≥3 points on histological examination.

We divided these 139 patients in two groups. One group was the construct group, which included 90 patients who received a histological examination from January 1990 to September 2011, and the other group was the validation group, which included 49 patients who received a histological examination from October 2011 to December 2013. The study was approved by the Institutional Review Board of our hospital.

### Definitions of hypertension and diabetes mellitus

Hypertension was defined as a seated systolic/diastolic blood pressure of >140/>90 mmHg measured after 5 min of rest [20]. Diabetes was diagnosed based on the 2010 criteria of the American Diabetes Association [21]. These criteria include: (1) casual plasma glucose ≥200 mg/dl; (2) fasting plasma glucose ≥126 mg/dl; and (3) 2-h post-glucose (oral glucose tolerance test) ≥200 mg/dl.

### Screening methods for viral hepatitis (hepatitis C and B virus)

Hepatitis C virus antibodies and hepatitis B surface antigen were examined at study entry. Hepatitis C virus antibodies were detected with a third-generation enzyme-linked immunosorbent assay (Abbott Laboratories, North Chicago, IL). Hepatitis B surface antigen was detected by radioimmunoassay (Abbott Laboratories).

### Histopathological examination of the liver

Liver biopsy specimens were obtained using a 14-gauge modified Vim Silverman needle (Tohoku University style, Kakinuma Factory, Tokyo, Japan), a 16-gauge core tissue biopsy needle (Bard Peripheral Vascular Inc., Tempe, AZ, USA) or surgical resection. Tissue was fixed in 10 % formalin, and sections were stained with hematoxylin-eosin, Masson trichrome, silver impregnation, and periodic acid-Schiff after diastase digestion. Fibrosis was scored using the five-grade scale proposed by Brunt et al. [22] as follows: stage 0, normal connective tissue; stage 1, pericellular or perivenular fibrosis in zone 3 (pericentral vein area); stage 2, zone 3 perisinusoidal/pericellular fibrosis with focal or extensive periportal fibrosis; stage 3, bridging or septal fibrosis; and stage 4, cirrhosis.

In this study, we defined histologically advanced fibrosis as NASH stages 3 and 4.

NAFLD activity was scored with an eight-grade scale, namely, the NAFLD activity score (NAS) proposed by Kleiner et al. [23] was the unweighted sum of the scores for steatosis (0–3), lobular inflammation (0–3), and ballooning degeneration (0–2).

#### Calculation of the APRI and prediction of advanced fibrosis

The APRI was calculated according to the following formula:

$$ \text{APRI} = \frac{{\text{AST\,level}\,\left( {/\text{ULN}*} \right)}}{{\text{Platelet\,count}\,\left( {10^{9} /{\text{L}}} \right)}} \times 100 $$

*ULN, AST upper level of normal (33 IU/L).

As previously reported, an APRI > 1.50 is predictive of advanced fibrosis (PPV, 88 %; NPV, 64 %) [12]. In association with the APRI, hepatic fibrosis was assessed with the Ishak fibrosis scoring system [24]. Advanced fibrosis was defined as an Ishak score of ≥3 (presence of occasional bridging fibrosis). However, recently, another investigator concluded that for patients with NAFLD or NASH, an APRI > 0.98 was more suitable for predicting advanced fibrosis (NASH stages 3 and 4) (sensitivity, 75 %; specificity, 86 %; PPV, 54 %; NPV, 93 %) [17].

#### Calculation of the NAFLD fibrosis score and prediction of advanced fibrosis

The NAFLD fibrosis score was calculated according to the following formula:

$$\begin{aligned}   {\text{NAFLD}}\;{\text{fibrosis score}}  &=  - 1.675\;{+ }\;0.037\; \times \;{\text{age }}\left( {{\text{years}}} \right){ +}0.094 \times {\text{BMI}}\left( {{\text{kg}}/{\text{m}}^{2} } \right) \\    &\quad { + }1.13 \times {\text{IFG}}*/{\text{diabetes }}\left( {{\text{yes}} = 1,{\text{ no}} = 0} \right){+}0.99 \times {\text{AST}}/{\text{ALT ratio}} \\    &\quad  - 0.013 \times {\text{platelet }}( \times 10^{9} /{\text{L}}) - 0.66 \times {\text{albumin}}\;\left( {{\text{g}}/{\text{dL}}} \right) \\  \end{aligned}$$

*Impaired fasting glucose (IFG), fasting blood glucose ≥110 mg/dL.

Hepatic fibrosis was scored based on the five-grade scale proposed by Brunt et al. [22]. Advanced fibrosis was defined as stages 3 and 4.

#### Calculation of the FIB-4 index and prediction of advanced fibrosis

The FIB-4 index was calculated according to the following formula:

$$ {\text{FIB4-index}} = \frac{{{\text{age}}\,\left({\text{year}} \right) \times {\text{AST}}\,{\text{level}}}}{{{\text{Platelet}}\,{\text{count}}\,\left({10^{9}/{\text{L}}} \right) \times {\text{ALT}}\,{\text{level}}^{1/2}}} $$

As previously reported, a FIB-4 index >3.25 is predictive of advanced fibrosis (PPV, 65 %; NPV, 83 %) [14]. However, Shah et al. [19] reported that a FIB-4 index > 2.67 was predictive of advanced fibrosis in NAFLD patients (PPV, 80 %; NPV, 83 %).

In association with the FIB-4 index, hepatic fibrosis was assessed with the Ishak fibrosis scoring system [24]. Advanced fibrosis was defined as an Ishak score of ≥4 (presence of marked bridging fibrosis) [14].

#### Calculation of the BARD score and prediction of advanced fibrosis

The following points are assigned to each variable making up the BARD scoring system: BMI ≥ 28 kg/m^2^, 1 point; AST/ALT ratio ≥0.8, 2 points; and presence of diabetes, 1 point. Thus, the scores range from 0 to 4. As previously reported, BARD scores of 2–4 are associated with an odds ratio for advanced fibrosis of 17 (PPV, 43 %; NPV, 96 %) [15]. A patient with a BARD score of 2–4 plus NASH stages 3 or 4 was considered to have advanced fibrosis.

#### Calculation of the NIKEI and prediction of advanced fibrosis

The NIKEI was calculated according to the following formula:

$$ \begin{aligned} \text{Logit}P & = \ln \left({P/1 - P} \right) = \text{}-24.214 + 0.225 \times \text{age}\,\left(\text{{years}} \right) + 0.056 \times \text{AST}\left(\text{{IU/L}} \right) \\ & \quad + 5.044 \times \text{AST/ALT\,ratio} + 3.631 \times \text{total\,bilirubin}\,\left(\text{mg}/\text{dL} \right) \\ \end{aligned} $$

As previously reported, a NIKEI ≥ 0.2294 is predictive of advanced fibrosis (PPV, 60 %; NPV, 98 %) [16]. In association with the NAFLD fibrosis score, hepatic fibrosis was scored based on the five-grade scale proposed by Brunt et al. [22]. Advanced fibrosis was defined as stages 3 and 4.

### Statistical analysis

Non-parametric procedures were employed for the analysis of background characteristics and laboratory data among patients in each stage, including the Kruskal–Wallis test and the *χ*² test. The Wilcoxon signed-rank test was used to compare the FSN pre and post biopsy. The normality of the distribution of the data was evaluated by the Kolmogorov–Smirnov one-sample test.

Because certain variables partly did not conform to a normal distribution, the natural logarithmic transformations of bilirubin, AST, ALT, GGT, triglyceride, ferritin, type IV collagen 7S and CK18 (M30) were also analyzed in the following calculation. The natural logarithmic transformation of the results yielded a normal distribution or a symmetrical distribution for each of the analyzed factors. After the procedures, the following multiple regression analysis became rationally robust against deviations from the normal distribution. To avoid introducing any variables that were mutually correlated into the model, we checked the interaction between all pairs of variables by calculating the variance of inflation factors. Of the highly correlated variables, less significant factors were removed from the viewpoint of multicollinearity.

Multivariate regression analysis was performed with data from the 90 patients from the construct dataset to generate construct data of the predicting function. We used a stepwise method for selection of informative subsets of explanatory variables in the model. A multiple regression coefficient and coefficient of determination were also taken into account in the selection of variables. Next, we validated the obtained predictive function using the data from the remaining 49 patients in the validation dataset. A *p* value of less than 0.05 with a two-tailed test was considered to be significant.

For evaluation of the efficiency and usefulness of the obtained function for the estimation of fibrosis, we compared various fibrotic scores for NASH, including the APRI, NAFLD fibrosis score, FIB-4 index, BARD score and NIKEI. In addition, to assess the accuracy of the new discriminant score in determining NASH advanced fibrosis (stages 3 and 4), we compared various fibrotic scores for NASH, including the APRI, NAFLD fibrosis score, FIB-4 index, BARD score, and NIKEI, and we calculated the sensitivity (Se) and specificity (Sp) for each value of each test, and then constructed receiver operating characteristic (ROC) curves by plotting the Se against (1 − Sp) at each value. The diagnostic performance of the scoring systems was assessed by analysis of the ROC curves. The most commonly used index of accuracy was the area under the ROC curve (AUROC), with values close to 1.0 indicating high diagnostic accuracy.

Data analysis was performed with SPSS software version 16.0 for Windows (SPSS, Chicago IL).

## Results

### Laboratory data for each fibrotic stage in the construct group

There were 50 males and 40 females with a median age of 50 years (range, 20–83 years) in the construct group. Laboratory data of these 90 patients are shown in Table 1. Although several individual items were well correlated with the severity of hepatic fibrosis, significant overlap in values was noted among stages 1–4 for the following: AST, platelet count, total cholesterol, LDL-cholesterol, ferritin, and type IV collagen 7S.

**Table 1:** Demographic, laboratory, and histological data of patients in the construct group

Laboratory data	Stage 1 (*n* = 43)	Stage 2 (*n* = 12)	Stage 3 (*n* = 30)	Stage 4 (*n* = 5)	*p* value
Gender, M:F	27:16	5:7	16:14	2:3	0.494
Age, years^a^	46 (20–69)	46.5 (23–59)	55.5 (28–83)	57 (45–75)	0.054
Body mass index, kg/m^2a^	25.2 (20.5–38.2)	27.7 (22.5–35.8)	27.5 (21.2–33.4)	24.3 (21.8–37.5)	0.279
Albumin, g/dL^a^	4.1 (3.6–5.4)	4.2 (3.6–4.9)	4.0 (3.0–5.0)	3.9 (3.5–4.9)	0.137
Total bilirubin, mg/dL^a^	0.8 (0.3–1.8)	0.65 (0.2–2.7)	0.9 (0.3–2.2)	0.8 (0.6–1.0)	0.701
AST, IU/L^a^	48 (23–270)	78 (25–139)	71 (31–198)	66 (25–139)	0.022
ALT, IU/L^a^	96 (30–299)	147 (15–303)	107.5 (24–312)	82 (22–145)	0.398
γ-GTP, IU/L^a^	75 (20–266)	55 (20–310)	79.5 (28–549)	108 (40–182)	0.331
Platelet count, × 10^9^/L^a^	245 (139–363)	232.5 (183–389)	194 (105–366)	114 (45–190)	<0.001
Diabetes mellitus, yes/no	11/32	3/9	8/22	3/2	0.431
Total cholesterol, mg/dL^a^	215 (125–280)	225.5 (166–370)	202 (160–285)	161 (101–239)	0.040
Triglyceride, mg/dL^a^	141 (51–346)	140.5 (81–457)	136.5 (42–610)	110 (71–355)	0.647
LDL-cholesterol, mg/dL^a^	136 (71–190)	151 (81–243)	126.5 (69–220)	88 (45–133)	0.029
HDL-cholesterol, mg/dL^a^	44 (31–82)	47 (31–74)	44.5 (14–79)	46 (5–106)	0.940
Ferritin, ng/mL^a^	212 (10–733)	214 (123–424)	309 (10–1472)	60 (10–39)	0.022
Type IV collagen 7S, ng/mL^a^	3.5 (2.6–5.4)	3.9 (2.6–5.6)	4.7 (3.4–8.9)	5.6 (3.4–8.0)	<0.001
CK18 (M30), U/L^a^	305 (83–3,049)	345 (186–1,622)	560.5 (160–2,132)	250 (170–525)	0.072
*Liver histology findings* ^b^					
Total NAFLD activity score^a^	4.0 (3.0–7.0)	5.0 (3.0–6.0)	5.0 (3.0–7.0)	3.0 (3.0–6.0)	0.165
Hepatocellular ballooning^a^	1.0 (1.0–2.0)	1.0 (1.0–2.0)	2.0 (1.0–2.0)	1.0 (1.0–2.0)	<0.001
Steatosis^a^	2.0 (1.0–3.0)	2.0 (1.0–3.0)	2.0 (1.0–3.0)	1.0 (1.0–2.0)	0.050
Lobular inflammation^a^	1.0 (0.0–2.0)	1.0 (1.0–3.0)	1.5 (0.0–3.0)	1.0 (1.0–3.0)	0.018

*ALT*  alanine aminotransferase, *AST* aspartate aminotransferase, *γ-GTP* gamma-glutamyl transpeptidase, *HDL* high-density lipoprotein, *IU* international units, *LDH* lactate dehydrogenase, *LDL* low-density lipoprotein, *NAFLD* non-alcoholic fatty liver disease, *U* units

^a^Expressed as median (range)

^b^Histological features; NAFLD activity score was assessed on a scale of 0–8, with higher scores indicating more severe disease (the components of this measure are steatosis [assessed on a scale of 0–3], lobular inflammation [assessed on a scale of 0–3], and hepatocellular ballooning [assessed on a scale of 0–2])

In contrast, with regard to the liver histological findings, significant differences in the degree of hepatocellular ballooning, steatosis, and lobular inflammation were observed among stages 1–4.

#### Regression function generated from the construct group

**Fig. 1:**
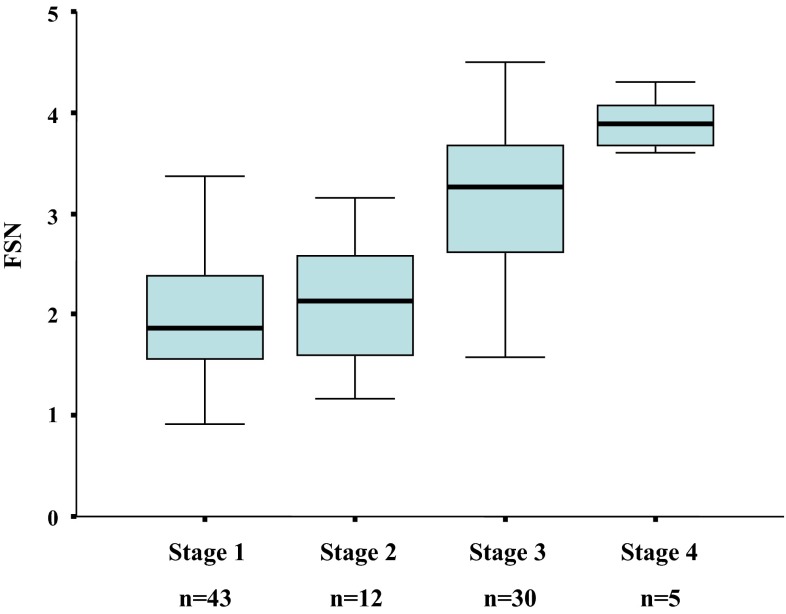
*Box* and *whisker plots* of the fibrotic score of patients with each stage of histological fibrosis in the construct dataset. The fibrotic score for NASH (FSN) was generated by the function, *z* = 1.022 × ln (type IV collagen 7S) (ng/mL) – 0.00680 × (platelet count) (×10^9^/L) + 1.925 × ln (AST) (IU/L) – 1.239 × ln (ALT) (IU/L) + 0.249

After stepwise variable selection, multivariate regression analysis ultimately obtained the following function: z = 1.022 × ln (type IV collagen 7S) (ng/mL) – 0.00680 × (platelet count) (×10^9^/L) + 1.925 × ln (AST) (IU/L) – 1.239 × ln (ALT) (IU/L) + 0.249. Median values of the FSN for stage 1 (*n* = 43), stage 2 (*n* = 12), stage 3 (*n* = 30) and stage 4 (*n* = 5) were calculated as 1.87, 2.14, 3.26 and 3.89, respectively (Fig. 1). The multiple regression coefficient and coefficient of determination were 0.70 and 0.46, respectively.

**Fig. 2:**
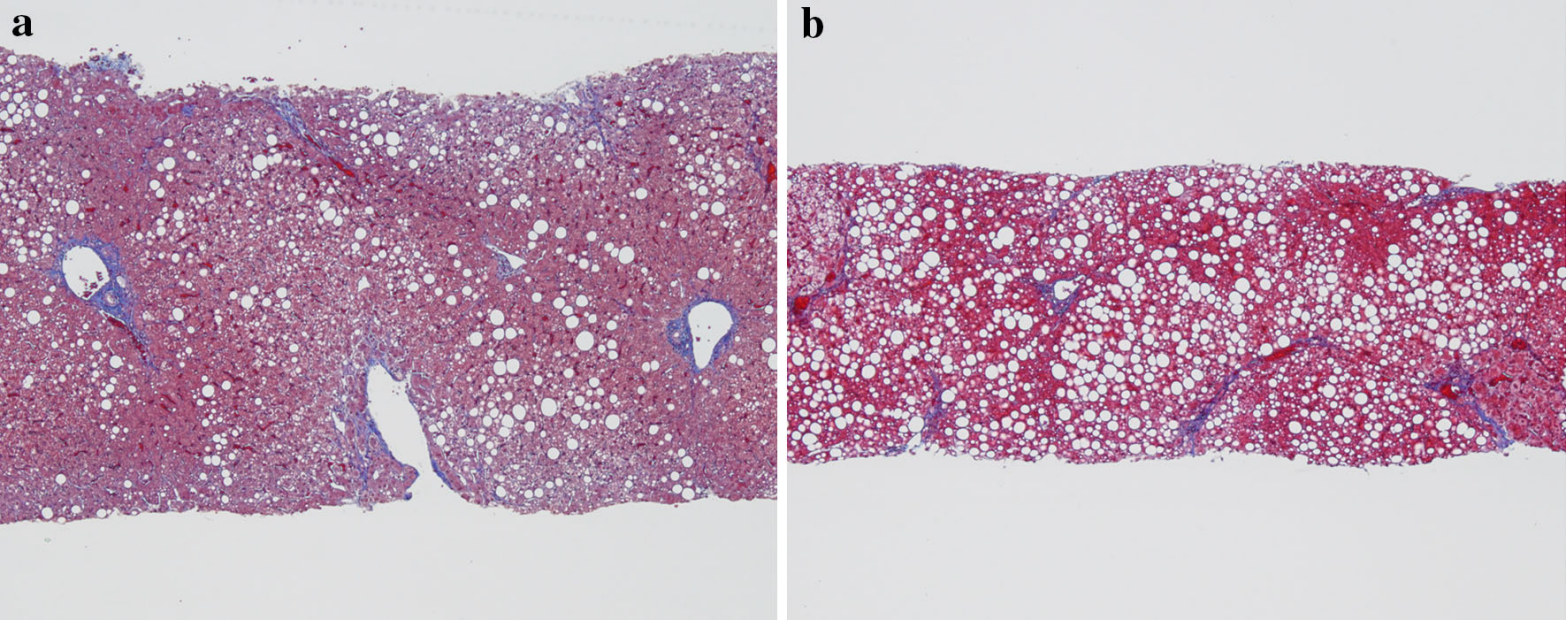
**a** A 51-year-old man with fibrotic stage 2 had a serum type IV collagen 7S concentration of 3.2 ng/mL, platelet count of 227 × 10^9^/L, AST 48 IU/L and ALT 89 IU/L. The regression function determined his fibrotic score as 1.78. (Masson trichrome staining of liver tissue; original magnification, 1 × 40). **b** Three years later, the same man underwent a repeat biopsy to assess disease control, and his fibrotic stage had progressed to 3, along with a serum type IV collagen 7S concentration of 4.0 ng/mL, platelet count of 236 × 10^9^/L, AST 122 IU/L and ALT 231 IU/L. The regression function determined his fibrotic score as 2.57, which was elevated. (Masson trichrome staining of liver tissue; original magnification, 1 × 40)

A 51-year-old man with fibrotic stage 2 (Fig. 2a) had a serum type IV collagen 7S concentration of 3.2 ng/mL, platelet count of 227 × 10^9^/L, AST 48 IU/L and ALT 89 IU/L. The regression function determined his fibrotic score as 1.78. Three years later, the same man underwent a repeat biopsy to assess disease control, and his fibrotic stage had progressed to 3 (Fig. 2b), along with a serum type IV collagen 7S concentration of 4.0 ng/mL, platelet count of 236 × 10^9^/L, AST 122 IU/L and ALT 231 IU/L. The regression function determined his fibrotic score as 2.57, which was elevated.

#### Validation of the discriminant function

**Fig. 3:**
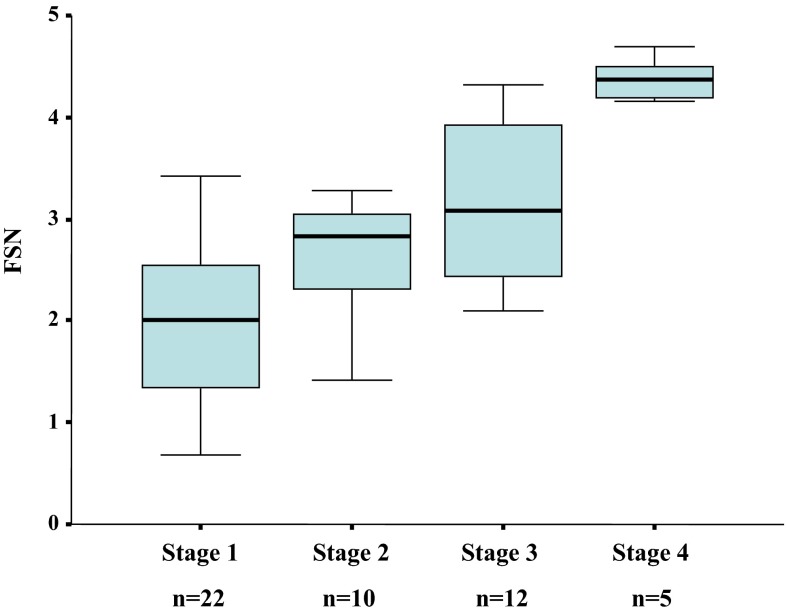
*Box* and *whisker plots* of the fibrotic score of patients with each stage of histological fibrosis in the validation dataset. The fibrotic score for NASH (FSN) was generated by the function, *z* = 1.022 × ln (type IV collagen 7S) (ng/mL) – 0.00680 × (platelet count) (×10^9^/L) + 1.925 × ln (AST) (IU/L) – 1.239 × ln (ALT) (IU/L) + 0.249

Laboratory data of the 49 patients in the validation group are shown in Table 2. When applying the regression function for the validation set, the FSN demonstrated good reproducibility, with a median score of 2.00 for stage 1 (*n* = 22), 2.83 for stage 2 (*n* = 10), 3.08 for stage 3 (*n* = 12) and 4.37 for stage 4 (*n* = 5) (Fig. 3).

**Table 2:** Demographic, laboratory and histological data of patients in the validation group

Laboratory data	Stage 1 (*n* = 22)	Stage 2 (*n* = 10)	Stage 3 (*n* = 12)	Stage 4 (*n* = 5)	*p* value
Gender, M:F	16:6	7:3	8:4	2:3	0.567
Age, years^a^	43 (30–73)	58 (38–72)	61.5 (38–76)	73 (67–85)	0.001
Body mass index, kg/m^2a^	28.7 (20.1–35.1)	25.4 (23.1–33.3)	28.5 (24.5–37.9)	24.4 (20.5–29.3)	0.150
Albumin, g/dL^a^	4.3 (3.7–4.6)	3.9 (3.5–4.3)	4.2 (3.7–4.7)	3.7 (3.4–4.4)	0.012
Total bilirubin, mg/dL^a^	0.9 (0.6–2.1)	0.8 (0.4–1.4)	0.9 (0.1–1.8)	0.9 (0.6–1.2)	0.847
AST, IU/L^a^	41 (19–164)	48 (37–92)	56 (24–150)	48 (41–84)	0.563
ALT, IU/L^a^	67 (31–275)	82 (57–213)	90 (29–238)	42 (28–48)	0.041
γ-GTP, IU/L^a^	83.5 (17–505)	51.5 (33–115)	58 (34–151)	58 (49–188)	0.195
Platelet count, × 10^9^/L^a^	245.5 (130–318)	176.5 (120–256)	195 (65–210)	115 (110–181)	<0.001
Diabetes mellitus, yes/no	2:20	1:9	6:6	1:4	0.031
Total cholesterol, mg/dL^a^	216.5 (101–270)	192 (153–276)	204.5 (144–265)	162 (105–171)	0.043
Triglyceride, mg/dL^a^	158.5 (31–570)	118.5 (69–314)	137 (63–252)	182 (36–330)	0.313
LDL-cholesterol, mg/dL^a^	121.5 (28–183)	115.5 (82–182)	118.5 (69–164)	68 (29–80)	0.019
HDL-cholesterol, mg/dL^a^	45 (32–76)	46 (33–65)	40.5 (32–68)	45 (27–50)	0.719
Ferritin, ng/mL^a^	357.5 (16–1432)	265.5 (55–1,149)	380.5 (108–1474)	200 (18–331)	0.186
Type IV collagen 7S, ng/mL^a^	3.7 (2.7–5.7)	4.65 (3.6–5.8)	5.25 (3.9–7.4)	6.9 (5.5–9.4)	<0.001
CK18 (M30), U/L^a^	279.5 (147–1,841)	405.5 (6–2,061)	541.5 (163–1,145)	441 (226–706)	0.476
*Liver histology findings* ^b^					
Total NAFLD activity score^a^	5.0 (3.0–7.0)	5.5 (3.0–8.0)	6.0 (3.0–7.0)	4.0 (3.0–6.0)	0.169
Hepatocellular ballooning^a^	1.0 (1.0–2.0)	1.5 (1.0–2.0)	2.0 (1.0–2.0)	2.0 (1.0–2.0)	0.027
Steatosis^a^	2.0 (1.0–3.0)	2.0 (1.0–3.0)	2.0 (1.0–3.0)	1.0 (1.0–2.0)	0.070
Lobular inflammation^a^	1.0 (1.0–2.0)	2.0 (1.0–3.0)	2.0 (1.0–2.0)	2.0 (1.0–3.0)	0.004

*ALT* alanine aminotransferase, *AST* aspartate aminotransferase, *γ-GTP* gamma-glutamyl transpeptidase, *HDL* high-density lipoprotein, *IU* international units, *LDH* lactate dehydrogenase, *LDL* low-density lipoprotein, *NAFLD* non-alcoholic fatty liver disease, *U* units

^a^Expressed as median (range)

^b^Histological features; NAFLD activity score was assessed on a scale of 0–8, with higher scores indicating more severe disease (the components of this measure are steatosis [assessed on a scale of 0–3], lobular inflammation [assessed on a scale of 0–3], and hepatocellular ballooning [assessed on a scale of 0–2])

#### Comparisons of efficacy with various fibrotic scores and to evaluate prediction power of NASH advanced fibrosis

To evaluate the efficacy and usefulness of the obtained FSN, we compared it with previously reported fibrotic scores using construct data. Spearman’s correlation coefficients for the APRI, NAFLD fibrosis score, FIB-4 index, BARD score and NIKEI were 0.462 (*p* < 0.001), 0.458 (*p* < 0.001), 0.578 (*p* < 0.001), 0.352 (*p* = 0.001) and 0.432 (*p* < 0.001), respectively. Our FSN showed a Spearman’s correlation coefficient of 0.685 (*p* < 0.001), which was a much higher value than the others.

**Fig. 4:**
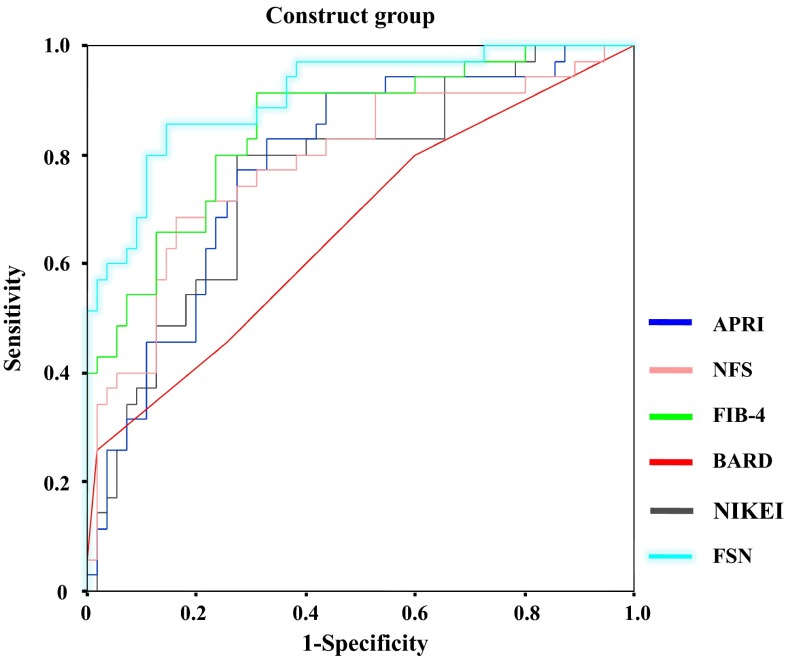
ROC curves for various fibrotic scores for NASH (FSN, APRI, NAFLD fibrosis score, FIB-4 index, BARD score and NIKEI) in the construct dataset. The area under the ROC curve (AUROC, 95 % CI) in the construct group was greatest for the FSN (0.909, 0.847–0.970), followed by the FIB-4 index (0.850, 0.769–0.932), NAFLD fibrosis score (0.786, 0.685–0.887), APRI (0.781, 0.683–0.878), NIKEI (0.758, 0.656–0.860) and BARD score (0.664, 0.547–0.782)

In addition, to evaluate the efficacy and usefulness of the obtained FSN to predict advanced fibrosis of NASH (stages 3–4), we compared it with previously reported fibrotic scores using training data (APRI, NAFLD fibrosis score, FIB-4 index, BARD score and NIKEI). The area under the ROC curve (AUROC, 95 % CI) in the construct group was greatest for the FSN (0.909, 0.847–0.970), followed by the FIB-4 index (0.850, 0.769–0.932), NAFLD fibrosis score (0.786, 0.685–0.887), APRI (0.781, 0.683–0.878), NIKEI (0.758, 0.656–0.860) and BARD score (0.664, 0.547–0.782) (Fig. 4).

#### Change in the FSN in patients with NASH who received repeat biopsy

In this study group, 25 of 139 NASH patients received a repeat biopsy, and their histological fibrotic stage had either been maintained or had progressed.

**Fig. 5:**
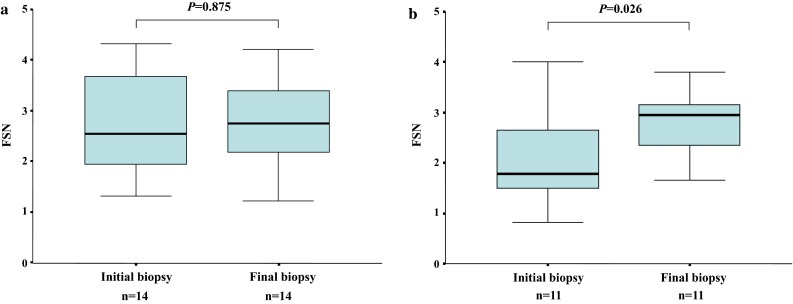
**a** Change in the FSN in 14 patients who maintained their histological fibrotic stage on repeat biopsy; there were no significant differences between FSNs at the initial and final biopsy (median FSN; 2.54 vs 2.74, respectively; *p* = 0.875). **b** Change in the FSN in 11 patients whose histological fibrotic stage had progressed on repeat biopsy; there were significant elevations between FSNs at the initial and final biopsy (median FSN; 1.79 vs 2.94, respectively; *p* = 0.026)

In 14 patients whose histological fibrotic stage had been maintained, there were no significant differences between the FSNs at the initial and final biopsy (median FSN; 2.54 vs 2.74, respectively; *p* = 0.875) (Fig. 5a).

In contrast, in the 11 patients whose histological fibrotic stage had progressed by the time of the repeat biopsy, there were significant elevations between the FSNs at the initial and final biopsy (median FSN; 1.79 vs 2.94, respectively; *p* = 0.026) (Fig. 5b).

## Discussion

Up to now, the definitive diagnosis of NASH has been based on histopathological evaluation. However, in Japan, many patients with NAFLD are diagnosed with NASH using US only, because liver biopsies have a risk of major complications, such as intraperitoneal bleeding. However, some noninvasive scoring systems (APRI, NAFLD fibrosis score, FIB-4 index, BARD score, and NIKEI) for predicting fibrosis have become available [13, 15–17, 19]. However, these studies were principally aimed at differentiation of advanced fibrotic stages of 3–4 from mild fibrotic stages of 1–2. Those discriminative functions were insufficient to recognize the stepwise progression of NASH from stage 1 through stage 4. This dichotomy (mild or severe) of NASH seemed less valuable in the study of disease progression, disease control abilities of new agents and estimation of histological improvement after dietary and kinesiology intervention. A histology-oriented, practical and reliable formula is therefore required for the diagnosis and investigation of NASH. This study aimed to establish a non-invasive evaluation and calculation of liver fibrosis for patients with NASH.

In this study, 139 patients with histologically proven NASH were analyzed. To obtain the most suitable equation approximating histological fibrotic stage, multivariate analysis was performed using two demographic parameters (age and sex) and 15 hematological and biochemical markers with or without logarithmic transformation.

Multiple regression analysis ultimately generated a first-degree polynomial function consisting of four variables: type IV collagen 7S, platelet count, AST and ALT. The obtained value of the FSN was generated to imitate actual histological staging. The FSN fit sufficiently to actual fibrotic stages with some overlap, as is usually found in histological ambiguity judged to be caused by the heterogeneity of fibrosis and sampling error stemming from a transitional histological staging. Considering the limitation of pathological difficulty in differentiation of the four continuous disease entities, the regression function obtained showed satisfactorily high accuracy rates in the prediction of liver disease severity.

The FSN seemed a very useful quantitative marker in evaluating the fibrotic severity of NASH patients without invasive procedures and without any specialized US or magnetic resonance imaging. The score can be calculated for any patient with NASH. Although this multiple regression model dealt with appropriate logarithmic transformation for non-normal distribution parameters, the regression analysis was based on a linear regression model. Very slight fibrosis can be calculated as less than 1.00, which is commonly found with a slight degree of steatohepatitis with a tiny fibrotic change as stage 0. Very severe fibrosis may be calculated as more than 4.00, which is an unimaginable and nonsense number in the scoring system of fibrosis.

In addition, to evaluate the efficacy and usefulness of the obtained FSN to predict advanced fibrosis of NASH (stages 3–4), we compared the FSN with previously reported fibrotic scores using training data (APRI, NAFLD fibrosis score, FIB-4 index, BARD score, and NIKEI). The AUROC, 95 % CI in the construct group was greatest for the FSN (0.909, 0.847–0.970), followed by the FIB-4 index (0.850, 0.769–0.932), NAFLD fibrosis score (0.786, 0.685–0.887), APRI (0.781, 0.683–0.878), NIKEI (0.758, 0.656–0.860), and BARD score (0.664, 0.547–0.782) respectively.

Therefore, our new discriminant score predicts the fibrotic stage of NASH well, and has good prediction power for detecting NASH advanced fibrosis.

In addition, the FSN is useful for long-term follow-up of NASH patients. The change in the FSN reflects the histological transition of fibrosis in NASH patients well (Fig. 5a, b). This provides an advantage for long-term follow-up of NASH patients in daily clinical practice.

However, this study has some limitations. First, this was a retrospective single-center cohort study that evaluated a small number of patients. A further large-scale study is needed to evaluate this discriminant score. Second, we could not compare our FSN and transient elastography in this study. Therefore, we must compare the diagnostic ability of the FSN and transient elastography in a future study. However, we believe that the impact of this new discriminant score on the routine clinical care of patients with NAFLD, especially NASH patients, will be enormous. We also think that the progression of many high-risk patients to advanced liver disease, including decompensated liver cirrhosis and hepatocellular carcinoma, will be prevented by early detection of disease progression using this discriminant score.

In conclusion, the FSN is a useful and reliable biomarker for the prediction of liver fibrosis in patients with NASH. The FSN is expected to be introduced and utilized in varied kinds of studies and trials. Its accuracy and reproducibility require further validation with larger numbers of patients in several countries other than Japan.

## Abbreviations

ALT: Alanine aminotransferase
AST: Aspartate aminotransferase
BMI: Body mass index
CI: Confidence interval
FSN: Fibrotic score for NASH
γ-GTP: Gamma-glutamyl transpeptidase
HDL: High-density lipoprotein
HCC: Hepatocellular carcinoma
LDH: Lactate dehydrogenase
LDL: Low-density lipoprotein
NAFLD: Non-alcoholic fatty liver disease
NASH: Non-alcoholic steatohepatitis
US: Ultrasonography
